# Study on the Synthesis of Castor Oil-Based Plasticizer and the Properties of Plasticized Nitrile Rubber

**DOI:** 10.3390/polym12112584

**Published:** 2020-11-03

**Authors:** Qinghe Fu, Jihuai Tan, Fang Wang, Xinbao Zhu

**Affiliations:** 1 College of Chemical Engineering, Nanjing Forestry University, Nanjing 210037, China; fqhtime@163.com (Q.F.); jihuaitan@njfu.edu.cn (J.T.); 2 Jiangsu Co-Innovation Center of Efficient Processing and Utilization of Forest Resources, Nanjing Forestry University, Nanjing 210037, China

**Keywords:** castor oil-based plasticizers, nitrile rubber, mechanical properties, aging resistance, thermal stability

## Abstract

A series of new environment-friendly plasticizers was synthesized from castor oil and used to plasticize nitrile rubber (NBR). The test results showed that tensile strength, elongation at break, and tear strength of NBR vulcanizates plasticized by castor oil-based plasticizers were found to be better than that of dioctyl phthalate (DOP). The aging test taken demonstrated that the castor oil-based plasticizers could improve the hot air and oil aging resistance of NBR vulcanizates. The thermal stability test illustrated that castor oil-based plasticizers enhanced the thermal stability of NBR vulcanizates, and the initial decomposition temperatures (T_10%_) were about 100 °C higher than that of DOP. In general, the studies manifested that EACO and EBCO can replace DOP to plasticize NBR and are used in fields that require high mechanical properties, aging resistance, and thermal stability. This study emphasizes the effects of sustainable, cost-effective, and high-efficiency plasticizers on NBR.

## 1. Introduction

Nitrile rubber (NBR) is an unsaturated copolymer prepared by the emulsion polymerization of acrylonitrile and butadiene; it offers good oil, chemical, and abrasion resistance. It is mainly used to manufacture various oil-resistant and sealing rubber products in the military, automotive, aviation, and petroleum industries [1,2,3,4]. However, the polar cyano structure of NBR results in high viscosity, which is unfavorable for processing and further use. Hence, it is necessary to add plasticizers to reduce the viscosity and improve its processability [4]. The plasticizers commonly used in NBR are mainly phthalate esters, which contain ester bonds that can form hydrogen bonds with the cyano groups in the NBR molecule to make plasticizers; thus, offering the copolymer good compatibility [5]. However, considering the direct and indirect threats of phthalate plasticizers to humans and the environment, it is necessary to explore alternative renewable plasticizers for nitrile rubber [6].

Over decades, the use of vegetable oil in the field of plasticizers has increased because of advantages such as wide distribution, non-toxicity, low price, environmental protection, and renewable offerings [7]. Vegetable oils contain fatty acid glyceride bonds, which are polar and highly compatible with NBR (high ACN content). Therefore, vegetable oils have great potential application value in NBR [5]. However, a lot of vegetable oils, such as soybean oil, linseed oil, palm oil, olive oil, etc., were used in NR [4,8], SBR [9,10,11], EPDM [12,13], etc., while its use in NBR was relatively less [14,15,16], which did not materialize the characteristics of vegetable oils naturally containing polar groups. Consequently, castor oil, with more inartificial polar groups, has attracted our attention.

Castor oil, known as oil grown on land, is an inedible oil mainly produced in Asia and Africa [17]. It is unique because it carries a special ricinoleic acid, which has an 18-carbon backbone with a hydroxy group on the 12-carbon atom and a cis double bond between carbons 9 and 10. The hydroxyl in castor oil molecule can undergo esterification to obtain more polar groups to improve its compatibility with polar materials such as PVC and NBR. Therefore, when compared to other vegetable oils, castor oil has more application potential in the plasticizer field. There are several studies on the plasticization of PVC with castor oil-based plasticizers [18,19,20], but very few on NBR.

In this study, five environmentally friendly castor oil-based plasticizers were prepared through simple esterification and epoxidation reactions, and used to plasticize NBR. The properties were investigated and compared to commercial plasticizer DOP in order to explore the possibility of replacing petrochemical-based phthalate plasticizers in NBR.

## 2. Experimental

### 2.1. Materials

Hydrogen peroxide, acetic anhydride, benzoyl chloride, acetic acid, calcium oxide, toluene, sodium hydroxide, sodium carbonate, hydrochloric acid, and acetone was provided by Nanjing Chemical Reagent Co., Ltd. (Nanjing, China); castor oil and cation exchange resin were provided by Anhui Xinyuan Chemical Co., Ltd. (Huangshan, China). NBR (3345C) was provided by LANXESS-TSRCChemical Industrial Co., Ltd. (Nantong, China).

### 2.2. Synthesis of Epoxy Castor Oil (ECO)

Accurately weighed quantitative castor oil, acetic acid, toluene, and cation exchange resin catalyst were added to a four-necked flask equipped with a stirrer, a thermometer, a constant pressure funnel, and a ball condenser; 30% hydrogen peroxide was added dropwise into the reaction after 2 h and stirred at 60 °C for 5 h. Then, the reaction mixture was filtered to recycle the cation exchange resin, and the remaining fluid was washed to be neutral with sodium carbonate solution and distilled water. The water and solvent were distilled under reduced pressure to obtain ECO. The epoxy value, hydroxyl value, viscosity, and glass transition temperature (Tg) of the synthetic castor oil-based plasticizers and DOP are given in Table 1.

### 2.3. Synthesis of Acetylated Castor Oil (ACO)

Accurately weighed quantitative castor oil and acetic anhydride was placed in a four-necked flask, and stirred at 140 °C for 2 h. The reaction mixture was then neutralized with sodium carbonate solution and distilled water. Water was removed by vacuum distillation.

### 2.4. Synthesis of Epoxy Acetylated Castor Oil (EACO)

Accurately weighed quantitative castor oil and acetic anhydride were placed in a four-necked flask and stirred at 140 °C for 2 h. The reaction mixture was then cooled to 60 °C, and toluene and cation exchange resin catalyst were added. 30% hydrogen peroxide was then added dropwise after 2 h and stirred for 5 h. Finally, the reaction mixture was treated as per the ECO preparation method to obtain EACO.

### 2.5. Synthesis of Benzoyl Castor Oil (BCO)

Accurately weighed quantitative castor oil, benzoyl chloride, calcium oxide, and toluene were placed in a four-necked flask and stirred at 110 °C for 3 h. The reaction mixture was then treated as per the ACO preparation method to gain BCO.

### 2.6. Synthesis of Epoxy Benzoyl Castor Oil (EBCO)

Accurately weighed quantitative BCO, acetic acid, toluene, and cation exchange resin catalyst were placed in a four-necked flask and heated. 30% hydrogen peroxide was added dropwise after 2 h and stirred at 60 °C for 5 h. The reaction’s post-treatment mixture was the same as that for the ECO preparation method to obtain EBCO.

### 2.7. Processing of Plasticized Compounds

The formulation of the materials (phr): NBR 100, carbon black N550 50, carbon black N770 50, plasticizer 20, ZnO 5, Stearic acid 1.2, antioxidant poly(1,2-dihydro-2,2,4-trimethyl-quinoline) (RD) 1.3, sulfur 1.2, accelerator tetramethylthiuram disulfide (TMTD) 0.5, accelerator *n*-cyclohexyl-benzothiazole-2-sulfonamide (CBS) 1.5.

First, raw NBR and carbon black (N550, N770) were blended on the two-roll mill (XK-160, Guangdong Zhanjiang Machinery Factory, Zhanjiang, Guangdong Province, China) at 25 ℃ for 5 min. Then, the plasticizer, ZnO, stearic acid, RD, TMTD, CBS, and S were added into the above NBR, respectively. The mixture was blended on the mill for about 40 min to gain homogeneous NBR compounds. Eventually, the NBR compounds were vulcanized through a compression molding press at 170 °C, 15 MPa. The NBR vulcanizates plasticized by different castor oil-based plasticizers were respectively named as NBR-DOP, NBR-ECO, NBA-ACO, NBA-EACO, NBA-BCO, and NBR-EBCO.

### 2.8. Tests

FT-IR spectra were acquired in the range of 400 to 4000 cm^−1^ recorded on the Nicolet FTIR-360 (Nicolet Instrument Crop., Madison, WI,, USA) Fourier transform infrared spectrophotometer. ^1^H NMR spectra were recorded by using an AVANE400 (Brucker Biospin Company, Fällanden, Switzerland) with deuterated chloroform as a solvent. The Mooney viscosity test of unvulcanized rubber was measured using the GT-7080S2 Mooney viscometer (Gotech Testing Machines (Dongguan) Co., Ltd., Dongguan, China) based on GB/T1232.1-2000. Cure characteristics were examined according to GB/T16584-1996 with M2000AN rotorless rheometer (Gotech Testing Machines (Dongguan) Co., Ltd., Dongguan, China). The temperature was set at 160 °C, and the time at 8 min. Hardness (Shore A) of the samples was determined as per GB/T531.1-2008. Tensile and tear strength were determined according to GB/T528-2009 and GB/T529-2008, respectively, using the AI-7000M microcomputer tensile testing machine (Gotech Testing Machines (Dongguan) Co., Ltd., Dongguan, China) with an applied load of 500 mm/min. The tensile strength, elongation at break, and tear strength were calculated from the average of 5 samples. Compression set (CS) values were determined according to GB/T7759.1-2015. Samples with 13 mm thickness and 29 mm diameter were compressed to constant strain (~25%) and kept at 100 °C for 72 h. For the hot air aging test, the samples were kept for 72 h at 100 °C. Finally, changes in material behavior were characterized by hardness, mass, volume, and tensile testing. According to GB/T1690-2010, the samples were immersed in ASTM 1 oil and then kept at 100 °C for 72 h. After that, the changes in material behavior of hardness, mass, volume, and tensile testing were characterized for oil aging resistance. The glass transition temperature (Tg) of the samples were measured by DSC (NETZSCH 214 POLYMA, Freistaat Bayern, Germany). The temperature was increased from −100 to 120 °C at a rate of 10 °C per minute. Thermogravimetric analysis of the samples was carried out on DTG-60AH TGA thermal analysis instruments (Netzsch Instrument Crop., Freistaat Bayern, Germany) in an N_2_ atmosphere (50 mL/min) at a heating rate of 10 °C/min from 30 to 900 °C. The fracture surface morphology of the NBR blends after tensile fracture was taken by the Hitachi S-4800 (Hitachi, Tokyo, Japan) scanning electron microscope (SEM) operated at 25 kV.

## 3. Results and Discussion

### 3.1. FTIR and NMR Characterization of the Synthesized Plasticizers

**Figure 1:**
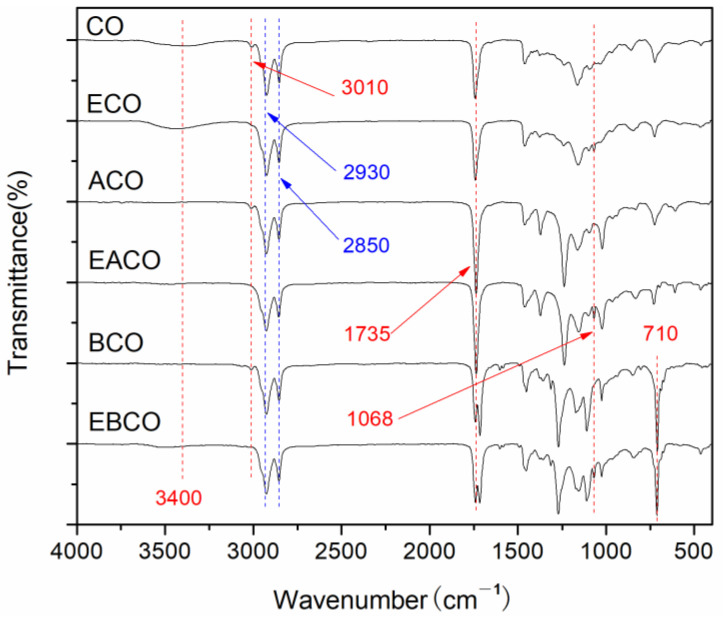
FT-IR spectra of CO and plasticizers synthesized.

The FT-IR spectra of CO, ECO, ACO, EACO, BCO, and EBCO are shown in Figure 1. In all the spectra curves, the methylene at 2930, 2850 cm^−1^ and carbonyl at 1735 cm^−1^ indicated the integrity of the main chain of the castor oil molecule. In the spectra of ACO, EACO, BCO, and EBCO, the hydroxyl group at 3400 cm^−1^ almost disappeared, illustrating that an acylation reaction had occurred. At 710 cm^−1^ is the bending vibration peak of the hydrocarbon in the monosubstituted benzene ring after benzoylation reaction. The carbon-carbon double bond of CO, ACO, and BCO at around 3010 cm^−1^ disappeared, while the epoxy group at 1068 cm^−1^ appeared in the spectra of ECO, EACO, and EBCO, demonstrating that the carbon-carbon double bonds were oxidized into epoxy groups.

**Figure 2:**
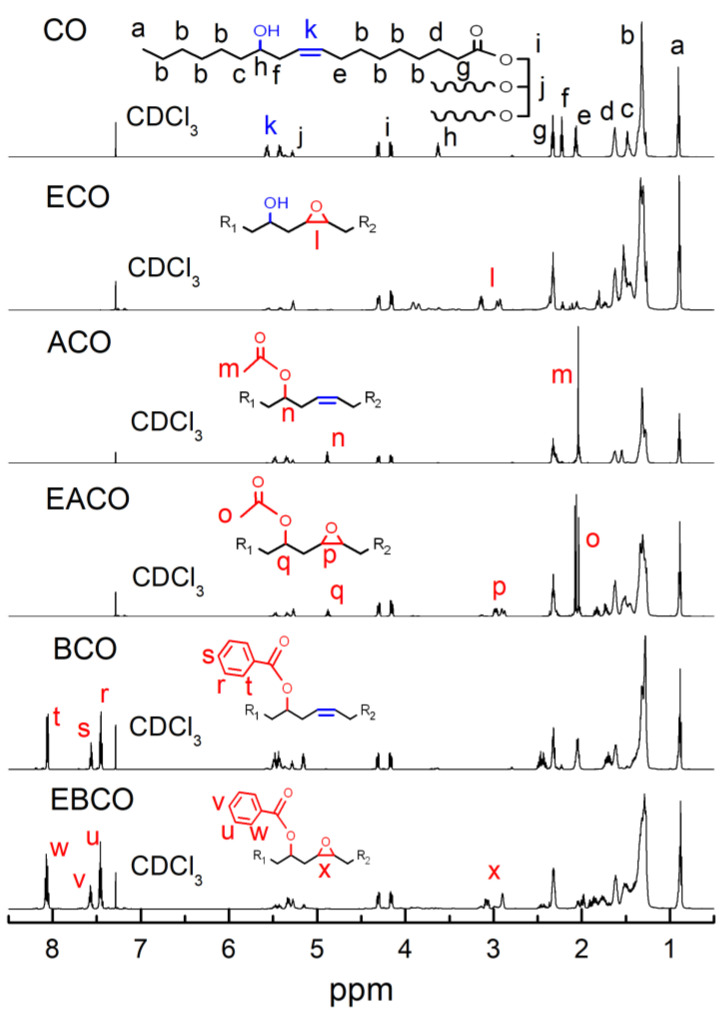
^1^H NMR spectrum of CO, ECO, ACO, EACO, BCO, and EBCO.

Figure 2 shows the ^1^H NMR of CO, ECO, ACO, EACO, BCO, and EBCO respectively. Compared to CO ^1^H NMR spectra, the signals in the 2.01 ppm of ACO and EACO spectra were associated with the shifted methyl hydrogen of the acetyl group and the proton signals in the 7.45–7.55 ppm and 8.1 ppm of BCO and EBCO spectra were associated with the hydrogen on the benzene ring, which demonstrated the acetylation and benzoylation reactions. The signals in the 5.45–5.55 ppm of ECO, EACO, and EBCO spectra were associated with weakening of the carbon-carbon double bands, while the signals in the 2.95–3.15 ppm were associated with the epoxy groups bands, which indicated that most of the carbon-carbon double bonds had been oxidized into epoxy groups during the epoxidation reaction.

### 3.2. Mooney Viscosity Test

**Figure 3:**
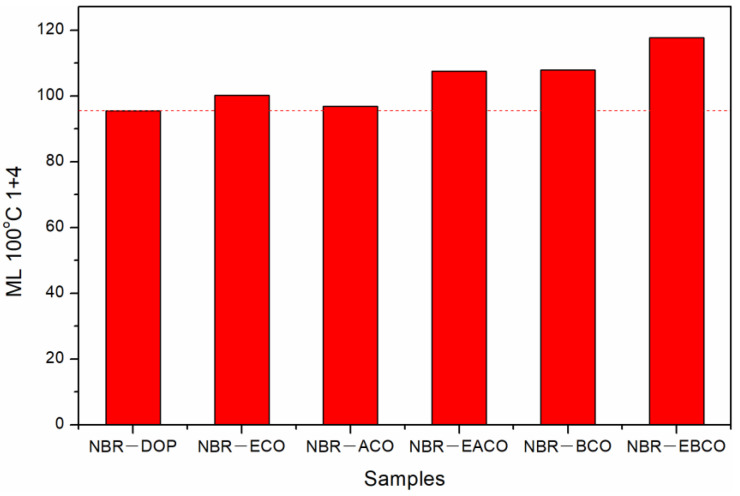
The Mooney viscosity of different NBR compounds.

The Mooney viscosity of different NBR compounds are given in Figure 3. The Mooney viscosity of the NBR-DOP compound was 95.52, while that of the NBR-ACO compound was 96.81, which meant that the plasticizing effect of the ACO plasticizing NBR compound was close to that of the DOP. The Mooney viscosity of NBR compounds plasticized by other four plasticizers was higher than that of DOP, indicating that the plasticizing efficiency is lower than DOP, which may be related to the viscosity of the plasticizer [21]. As shown in Table 1, the viscosity of DOP was 80, and the viscosities of ACO, EACO, BCO, and EBCO increased sequentially, as did the NBR compounds. Nevertheless, ECO was an exception, mainly because of the hydroxyl groups in ECO, which improved the compatibility of the plasticizer and NBR, and reduced the Mooney viscosity of the mixed rubber.

### 3.3. Cure Characteristics

In the production of rubber molding products, machinability research is very important, which can be determine vulcanization characteristics, such as maximum torque MH, minimum torque ML, scorch time t_c10_, and positive vulcanization time t_c90_ [22]. The vulcanization characteristics data of different NBR compounds are summarized in Table 2. It can be seen that the MH and ML of NBR vulcanizates plasticized by different plasticizers have little difference, and the change is similar to the Mooney viscosity. The t_c10_ represents the operating time of the compound and the T_c10_ of NBR-DOP was 1.57 min. The t_c10_ of the NBR-ACO and NBR-BCO compounds were close to NBR-DOP, while NBR-ECO and NBR-EACO containing epoxy groups were longer, which may be due to the presence of epoxy groups promoting the crosslinking of NBR and the vulcanizing agent during the vulcanization induction period. The positive vulcanization time t_c90_ represents the processing time of the compound in the molding production, and the t_c90_ of NBR-DOP was 2.97 min. The t_c90_ of the NBR-ACO and NBR-BCO compounds were much higher than that of NBR-DOP, while that of the NBR-ECO, NBR-EACO, and NBR-EBCO compounds reduced, which demonstrated that the existence of epoxy groups promotes the vulcanization process of NBR.

### 3.4. Physical and Mechanical Properties

The hardness, compression set, tensile strength, elongation at break, stress at 100% strain and tear strength of NBR vulcanizates are given in Table 3. The tensile strength and elongation at break of NBR-DOP were 17.18 MPa and 240.25%. The tensile strength and elongation at break of NBR vulcanizates plasticized by five castor oil-based plasticizers were higher than those of NBR-DOP, which indicated that the NBR spline plasticized by the synthetic castor oil-based plasticizers were more resistant to tensile failure than DOP, and the stretch effect was better than DOP. The higher tensile strength may be because the castor oil molecules have polar groups such as ester bonds and epoxy groups compared to DOP molecules, which increases the force between the molecules and the cohesive force of the rubber compounds. At the same time, the interaction between the rubber macromolecular chains and fillers were reduced, which led to improvements in the plasticizing effect. On the other hand, the long molecular chain of castor oil also increases the ductility of the NBR spline. The tear strength of the NBR-DOP spline was 57.94 MPa, while the tear strength of other five NBR splines were higher than that of NBR-DOP, which manifested that castor oil-based plasticizers can improve the ability of NBR to resist crack expansion and cracking. The hardness of NBR-EACO, NBR-BCO, and NBR-EBCO were found to be higher than that of NBR-DOP, showing that they have a higher crosslinking density. The compression set of NBR-DOP was 38.72%, while NBR-EACO, NBR-BCO, and NBR-EBCO were 38.47%, 38.52% and 39.14%, respectively, which represented that these three plasticizers have possible applications in rubber sealing products. However, the compression set of NBR-ECO and NBR-ACO was much higher than that of NBR-DOP, reaching 57.68% and 49.85%, which meant that the recovery ability of these two NBR products was weak.

### 3.5. Hot Air Aging Properties

**Figure 4:**
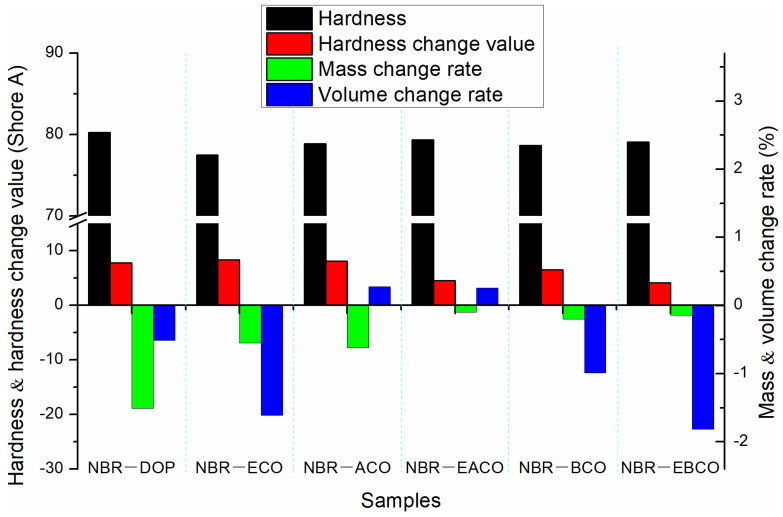
The change in hardness, mass, and volume of NBR vulcanizates after hot air aging.

The NBR vulcanizates were aged for 72 h in a circulating hot air environment at 100 °C, and then changes in hardness, mass, and volume of the samples were measured as shown in Figure 4; the mechanical properties after aging are listed in Table 4. The mass, volume, and hardness changes of the NBR-DOP vulcanizate after hot air aging were −1.51%, −0.51% and 7.8 A, respectively. Compared with NBR-DOP, the mass loss of vulcanizates plasticized by five castor oil-based plasticizers were less, which shown that the compatibility of the five castor oil-based plasticizers with NBR is better than that of DOP. This is because the synthetic plasticizers contain a large number of ester bonds and epoxy groups, which make plasticizers have more compatibility with NBR than DOP. Based on the mass, volume, and hardness change data, NBR-EACO had better heat-resistant air aging performance. The change rates of mass, volume, and hardness were −0.10%, 0.26% and 4.5 A, respectively, and less than that of NBR-DOP. After hot air aging, the tensile strength of the vulcanizates increased, but the elongation at break decreased significantly. This was mainly because during the hot air aging process, the samples continued to be vulcanized and crosslinked [23]. The change rates of tensile strength and elongation at break of NBR-DOP were 10.48% and −46.50%, while other vulcanizates had a smaller change in elongation at break and a wide variation in tensile strength. In general, the hot air aging performance of the NBR vulcanizates containing epoxy group plasticizers was significantly better than that of DOP.

### 3.6. Oil Aging Resistance

**Figure 5:**
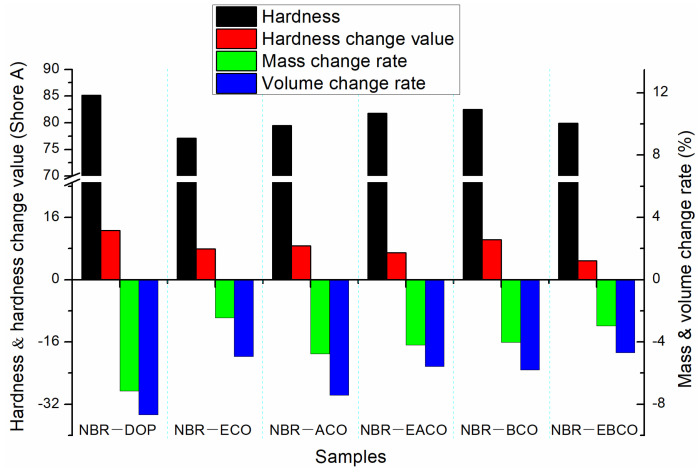
The change in hardness, mass, and volume of NBR vulcanizates after hot oil aging.

After immersing the NBR vulcanizate samples in ASTM 1 oil and aging at 100 °C for 72 h, the changes in hardness, mass, and volume of the samples are shown in Figure 5. The mass and volume of the NBR vulcanizates after hot oil aging varied greatly, which was because the plasticizers migrated to the oil when the samples were immersed in the polar ASTM 1 oil. The changes of NBR-DOP in mass, volume, and hardness after hot oil aging were −7.15%, −8.68%, and 12.7 A. The mass, volume, and hardness changes of vulcanizates plasticized by five castor oil-based plasticizers were smaller than those of NBR-DOP, which indicated that the NBR vulcanizates containing castor oil-based plasticizers have better oil migration resistance. According to Table 5, the tensile strength of the vulcanizates increased, but the elongation at break greatly reduced. This was mainly because in the process of hot oil aging, there also was the migration of plasticizers in addition to the thermal cracking of the crosslinking network. The change rates of tensile strength and elongation at break of NBR-DOP were 15.48% and −46.51%. Meanwhile, the variation of mechanical properties of other samples were almost less than that of NBR-DOP. All the oil aging properties manifested that castor oil-based plasticizers can improve the thermal oil aging stability of NBR vulcanizates.

### 3.7. Glass Transition Temperature

**Figure 6:**
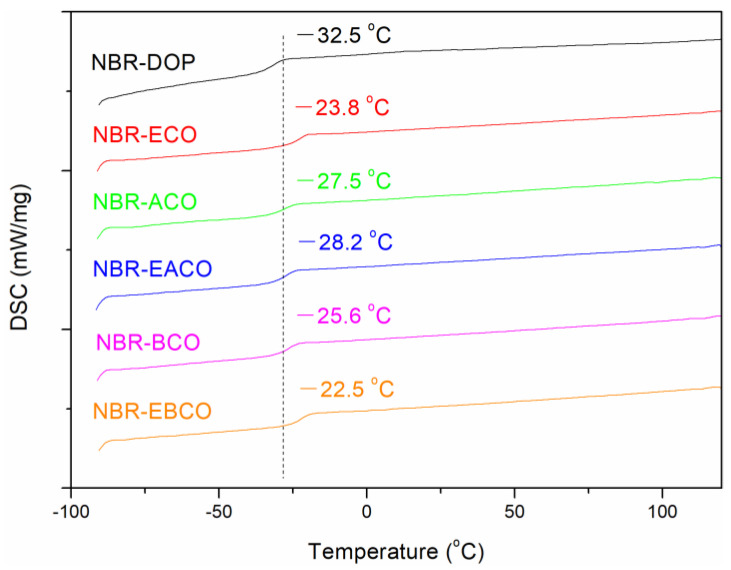
The glass transition temperature curves of NBR vulcanizates.

The glass transition temperature (Tg) of NBR vulcanizates was measured by DSC; the DSC thermograms are presented in Figure 6. Obviously, the DSC thermogram of every sample exhibited an inflection point, which showed that the plasticizers were compatible with NBR. The Tg of NBR-DOP was −32.5 °C, while the samples containing castor oil-based plasticizers were slightly higher than that of DOP. This might be due to the higher Tg of the castor oil-based plasticizers than DOP (shown in Table 1, except ACO, −73.3 °C). On the other hand, the castor oil-based plasticizers contained carbon-carbon double bonds and epoxy groups, which might participate in the vulcanization process. Therefore, the plasticizers were distributed in the vulcanizates in the state of chemical crosslinking [16].

### 3.8. Thermogravimetric Analysis

**Figure 7:**
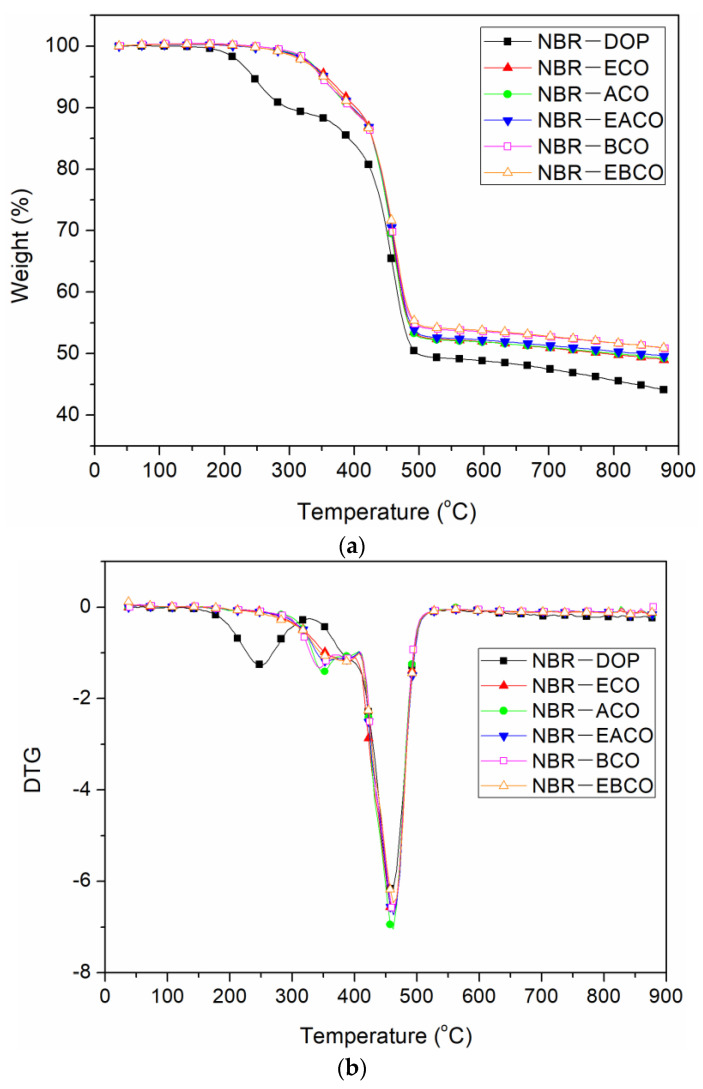
TGA curve (**a**) and DTG curve (**b**) of NBR vulcanizates.

The TGA curves of different NBR vulcanizates are shown in Figure 7 and the thermal performance data, including the weight loss of 10% (T_10%_), weight loss of 50% (T_50%_), and the residue at 900 °C are summarized in Table 6. The thermal weight loss of each vulcanizate was divided into two sections. The first section at 300–400 °C was the volatilization and decomposition of small molecules such as plasticizers, and the second section at 400–500 °C was the decomposition of the polymer molecular chain [24]. The first thermal weight loss temperature of NBR-DOP was 297.3 °C, while the NBR vulcanizates containing castor oil-based plasticizers had a peak temperature of 390–405 °C, which was about 100 °C higher than that of NBR-DOP. The higher temperature of initiation of degradation of these mixes was possibly because there were more physical and chemical crosslink points between the synthetic castor oil-based plasticizer and NBR, which was attributed to the interaction between the oils and NBR [15,25]. On the other hand, the castor oil-based plasticizers have a large molecular weight and a high vaporization point then that of DOP. It can be seen from Table 6 that the second thermal weight loss peak temperature of each vulcanizate was relatively close, and NBR-DOP was only about 5 °C lower than that of the other samples, which was mainly due to the heat resistance of the ester bonds in the castor oil-based plasticizers. At 900 °C, the residue of NBR-DOP was about 4–6% lower than others, which demonstrated that castor oil-based plasticizers can improve the thermal stability of NBR.

### 3.9. Fracture Surface Morphology Analysis

**Figure 8:**
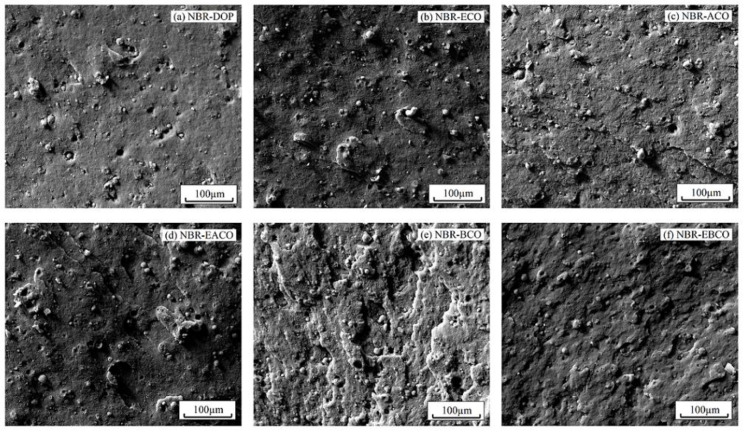
SEM images of (**a**) NBR-DOP, (**b**) NBR-ECO, (**c**) NBR-ACO, (**d**) NBR-EACO, (**e**) NBR-BCO and (**f**) NBR-EBCO.

The fracture surface morphology of NBR blends after tensile fracture are exhibited in Figure 8. Scanning electron microscopy provides direct evidence to evaluate the effect of plasticizers on the dispersion of carbon black. In Figure 8a, it can be seen that the addition of DOP slightly improved the dispersion of carbon black particles and the adhesion between carbon black particles and matrix NBR (the domain size range from 5.72 to 15.16 μm). However, for NBR-ECO (Figure 8b), carbon black particles seemed to be packed into agglomerates of smaller size between 5.37 and 13.68 μm, which indicated that the polar groups in the plasticizers promoted the dispersibility of carbon black particles in NBR. When compared to ACO and BCO, the carbon black particles in NBR-EACO and NBR-EBCO were smaller in size and had more even distribution. This suggested that the presence of polar groups made castor oil-based plasticizers act as interfacial agents, reducing the interfacial tension and, consequently, a reduction in particle size [26,27].

## 4. Conclusions

Five new environment-friendly plasticizers were synthesized using castor oil as raw material. The synthetic plasticizers were used to plasticize NBR and then compared to commercial plasticizer DOP. The mechanical properties of the vulcanizates illustrated that tensile strength, elongation at break, and tear strength of the NBR vulcanizates plasticized by castor oil-based plasticizers were all found to be improved, when compared to DOP. The aging test demonstrated that the castor oil-based plasticizer could improve the hot air and oil aging resistance of NBR vulcanizates. Thermal stability tests showed that castor oil-based plasticizers enhanced the thermal stability of NBR vulcanizates, and their initial decomposition temperature T_10%_ was about 100 °C higher than that of DOP. In summary, EACO and EBCO had better physical, mechanical, and aging resistance properties, and they can replace DOP in plasticizing NBR in fields that require high mechanical properties, aging resistance, and thermal stability.

## Figures and Tables

**Table 1:** Physical properties of plasticizers.

Plasticizers	Epoxy Value (mol/100 g)	Hydroxyl Value (mKOH/g)	Viscosity at 25 °C (mPa.s)	Tg (°C)
DOP	—	—	80	−71.6
ECO	0.23	—	2850	−52.2
ACO	—	10.06	150	−73.3
EACO	0.22	—	450	−64.5
BCO	—	12.99	500	−60.1
EBCO	0.18	—	2200	−45.2

**Table 2:** Vulcanization characteristics data of different NBR compounds.

Samples	MH (Nm)	ML (Nm)	t_c10_ (min)	t_c90_ (min)
NBR-DOP	23.27	2.16	1.57	2.97
NBR-ECO	19.61	2.50	1.28	2.23
NBR-ACO	19.42	2.55	1.53	3.68
NBR-EACO	21.48	2.57	1.43	2.60
NBR-BCO	21.40	2.61	1.60	4.72
NBR-EBCO	23.62	2.73	1.50	2.62

**Table 3:** Physical and mechanical properties of NBR vulcanizates.

Samples	TS_a_ (MPa)	EB (%)	S100 (MPa)	TS_b_ (N/mm)	H (Sh A)	CS (%)
NBR-DOP	17.18 ± 0.68	240.25 ± 19.16	6.56 ± 0.26	57.94 ± 2.36	72.5	38.72
NBR-ECO	17.48 ± 0.23	289.40 ± 11.00	6.04 ± 0.18	63.12 ± 1.16	69.2	57.68
NBR-ACO	17.52 ± 0.41	272.62 ± 11.68	6.50 ± 0.36	58.06 ± 2.58	70.8	49.85
NBR-EACO	17.66 ± 0.54	256.37 ± 12.63	7.22 ± 0.23	60.43 ± 2.34	74.9	38.47
NBR-BCO	18.30 ± 0.72	245.22 ± 10.64	7.12 ± 0.22	61.91 ± 1.88	73.2	38.52
NBR-EBCO	18.94 ± 0.43	257.73 ± 3.51	7.78 ± 0.33	63.89 ± 2.18	75.0	39.14

TS_a_—tensile strength, EB—elongation at break, S100—stress at 100% strain, TS_b_—tear strength, H—hardness, CS—compression set.

**Table 4:** The mechanical properties of NBR vulcanizates after hot air aging.

Sample	TS (MPa)	EB (%)	TSCR (%)	EBCR (%)
NBR-DOP	18.98 ± 0.99	128.54 ± 9.80	10.48	−46.50
NBR-ECO	18.70 ± 0.96	185.63 ± 16.35	6.98	−35.86
NBR-ACO	19.64 ± 0.56	153.59 ± 6.36	12.10	−43.66
NBR-EACO	19.10 ± 1.06	159.60 ± 17.97	8.15	−36.42
NBR-BCO	20.60 ± 1.35	158.80 ± 8.42	12.57	−35.24
NBR-EBCO	20.60 ± 0.74	168.43 ± 11.87	8.76	−34.65

TS—tensile strength, EB—elongation at break, TSCR—tensile strength change rate, EBCR—elongation at break change rate.

**Table 5:** The mechanical properties of NBR vulcanizates after hot oil aging.

Samples	TS (MPa)	EB (%)	TSCR (%)	EBCR (%)
NBR-DOP	19.84 ± 1.19	128.52 ± 13.14	15.48	−46.51
NBR-ECO	18.82 ± 0.26	190.55 ± 4.62	7.67	−34.16
NBR-ACO	19.00 ± 0.68	154.02 ± 7.78	8.45	−43.50
NBR-EACO	19.82 ± 0.92	155.23 ± 3.07	12.23	−38.16
NBR-BCO	19.04 ± 1.93	123.33 ± 17.40	4.04	−49.71
NBR-EBCO	20.40 ± 1.21	147.20 ± 13.06	7.71	−42.89

TS—tensile strength, EB—elongation at break, TSCR—tensile strength change rate, EBCR—elongation at break change rate.

**Table 6:** TGA data of NBR vulcanizates.

Sample	T_10%_ (°C)	T_p1_ (°C)	T_p2_ (°C)	Residue (%)
NBR-DOP	297.3	252.3	457.3	44.12
NBR-ECO	402.1	382.1	462.1	48.99
NBR-ACO	392.1	347.1	462.1	49.26
NBR-EACO	392.4	352.4	462.4	49.56
NBR-BCO	394.2	344.4	464.4	50.81
NBR-EBCO	392.0	387.0	462.0	50.93
